# The complete genome sequence of *Eimeria tenella* (Tyzzer 1929), a common gut parasite of chickens

**DOI:** 10.12688/wellcomeopenres.17100.1

**Published:** 2021-09-09

**Authors:** Eerik Aunin, Ulrike Böhme, Damer Blake, Alexander Dove, Michelle Smith, Craig Corton, Karen Oliver, Emma Betteridge, Michael A. Quail, Shane A. McCarthy, Jonathan Wood, Alan Tracey, James Torrance, Ying Sims, Kerstin Howe, Richard Challis, Matthew Berriman, Adam Reid

**Affiliations:** 1 Wellcome Sanger Institute, Cambridge, CB10 1SA, UK; 2 Royal Veterinary College, Hatfield, AL9 7TA, UK

**Keywords:** Eimeria tenella, Apicomplexa, parasite, protist, genome sequence, chromosomal

## Abstract

We present a genome assembly from a clonal population of *Eimeria tenella* Houghton parasites (Apicomplexa; Conoidasida; Eucoccidiorida; Eimeriidae). The genome sequence is 53.25 megabases in span. The entire assembly is scaffolded into 15 chromosomal pseudomolecules, with complete mitochondrion and apicoplast organellar genomes also present.

## Species taxonomy

Eukaryota; Apicomplexa; Conoidasia; Eucoccidiorida; Eimeriidae; Eimeria; *Eimeria tenella* Tyzzer 1929 (NCBItxid:5802).

## Introduction

The genome of *Eimeria tenella* (Houghton strain) was sequenced as part of the Darwin Tree of Life Project, a collaborative effort to sequence all of the named eukaryotic species in Britain and Ireland. Here we present a chromosomally complete genome sequence based on a clonal specimen maintained initially at the Houghton Poultry Research Station (HPRS) and more recently at the Royal Veterinary College, Hertfordshire, UK, where it was collected from experimentally infected *Gallus gallus domesticus*. This apicomplexan parasite is a major cause of coccidiosis in farmed chickens in the UK.

## Genome sequence report

**Figure 1.**
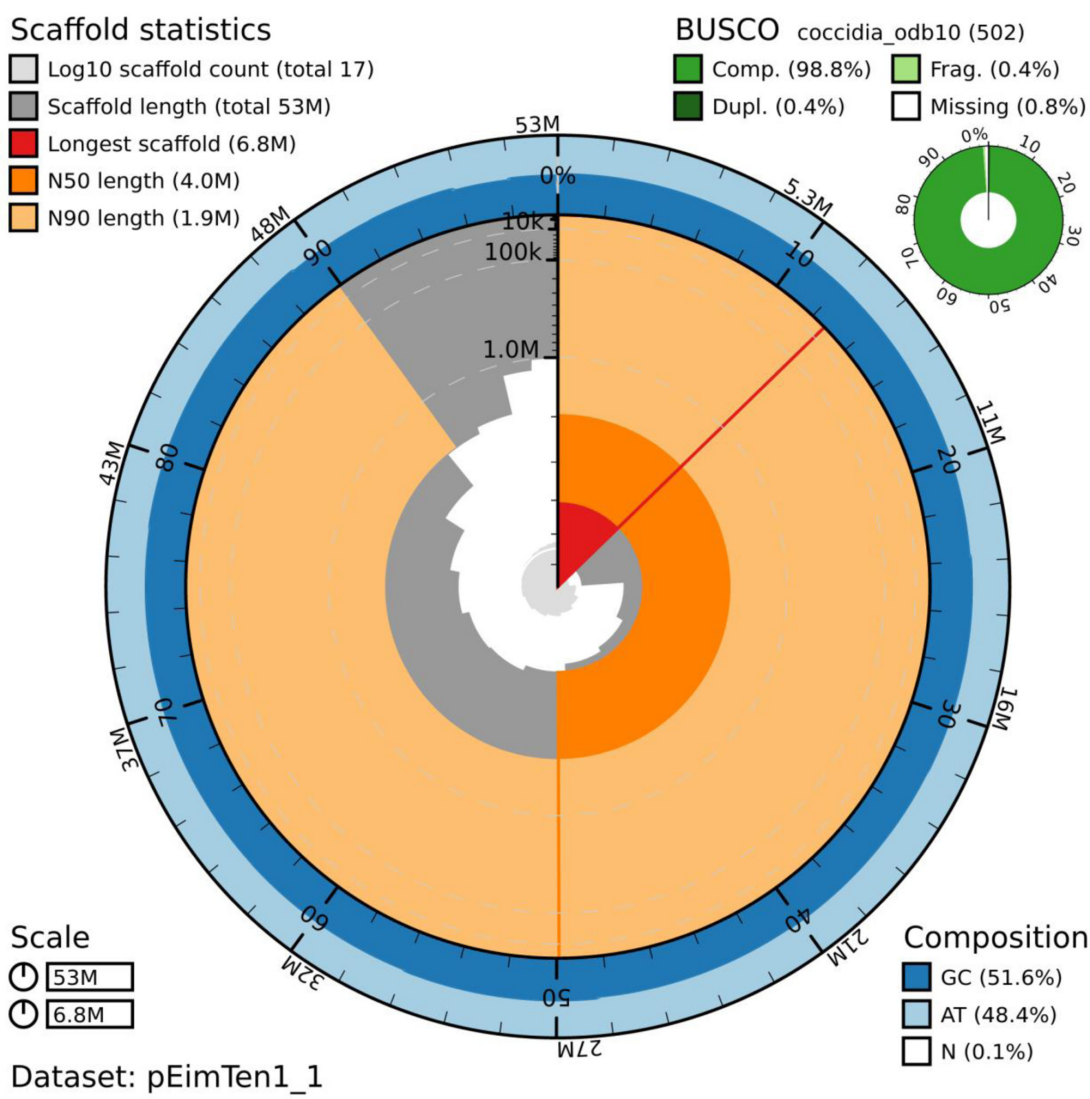
Genome assembly of *Eimeria tenella* Houghton, pEimTen1.1: metrics. The BlobToolKit Snailplot shows N50 metrics and BUSCO gene completeness. An interactive version of this figure is available at https://blobtoolkit.genomehubs.org/view/pEimTen1.1/dataset/pEimTen1_1/snail.

The genome was sequenced from a clonal specimen of *E. tenella* collected from experimentally infected *G. gallus domesticus* at the Royal Veterinary College, UK. A total of 41-fold coverage in Pacific Biosciences single-molecule long reads (N50 8 kb) and 107-fold coverage in 10X Genomics read clouds were generated. Primary assembly contigs were scaffolded with chromosome conformation Hi-C data. Manual assembly curation corrected 200 missing/misjoins, reducing the scaffold number by 77.9%, increasing the scaffold N50 by 0.1% and decreasing the assembly length by 1.85%. The final assembly has a total length of 53.25 Mb in 15 chromosomal scaffolds, one mitochondrial scaffold and one apicoplast scaffold. The total scaffold N50 was 4.01 Mb ( Table 1). The chromosomal scaffolds are numbered by sequence length, 1 being the smallest and 15 the largest, as is typical for Apicomplexa ( Figure 1– Figure 3; Table 2). The organellar mitochondrial and apicoplast genome sequences were each assembled into single contigs and circularized to remove redundancy. The assembly has a BUSCO v5.1.2 ( Simao *et al*., 2015) completeness of 98.8% and duplication rate of 0.2% using the coccidia_odb10 reference set.

**Table 1.** Genome data for *Eimeria tenella*, pEimTen1.1.

*Project accession data*	
Assembly identifier	pEimTen1.1
Species	*Eimeria tenella*
Specimen	pEimTen1
NCBI taxonomy ID	NCBI:txid5802
BioProject	PRJEB43184
BioSample ID	SAMEA7524401
Isolate information	Clonal specimen, Houghton strain
*Raw data accessions*	
PacificBiosciences SEQUEL I	ERR6447337
10X Genomics Illumina	ERX5693366-ERX5693369
Hi-C Illumina	ERX5693901
*Genome assembly*	
Assembly accession	GCA_905310635.1
Span (Mb)	381
Number of contigs	35
Contig N50 length (Mb)	14
Number of scaffolds	33
Scaffold N50 length (Mb)	14
Longest scaffold (Mb)	16
BUSCO * genome score	C:98.8%[S:98.4%,D:0.4%],F:0.4%, M:0.8%,n:502

*BUSCO scores based on the coccodia_odb10 BUSCO set using v5.1.2. C= complete [S= single copy, D=duplicated], F=fragmented, M=missing, n=number of orthologues in comparison. A full set of BUSCO scores is available at https://blobtoolkit.genomehubs.org/view/Eimeria%20tenella/dataset/pEimTen1_1/busco.

**Figure 2.**
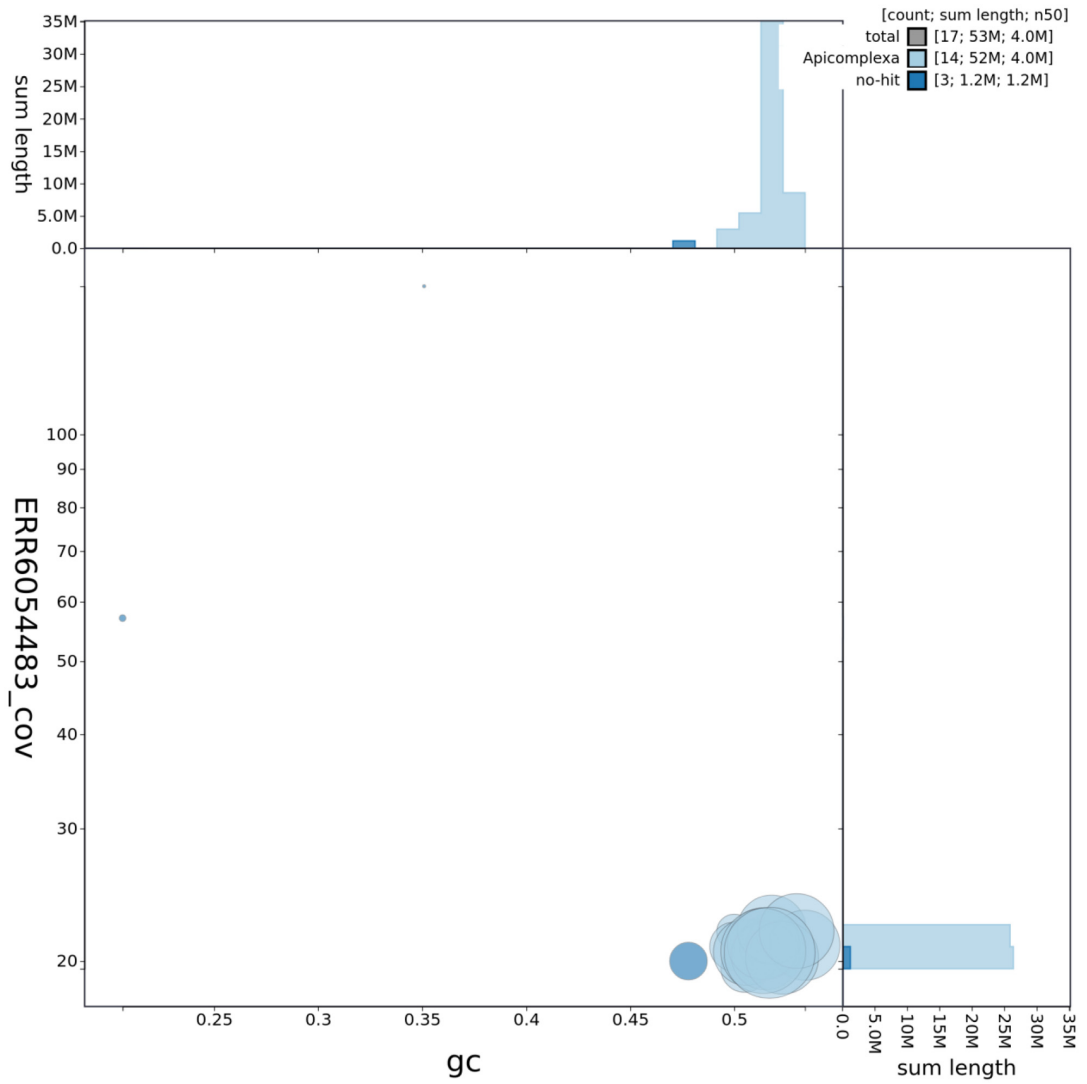
Genome assembly of *Eimeria tenella* Houghton, pEimTen1.1: GC coverage. BlobToolKit GC-coverage plot. Scaffolds are coloured by phylum. Circles are sized in proportion to scaffold length. Histograms show the distribution of chromosome length sum along each axis. An interactive version of this figure is available at https://blobtoolkit.genomehubs.org/view/pEimTen1.1/dataset/pEimTen1_1/blob.

**Figure 3.**
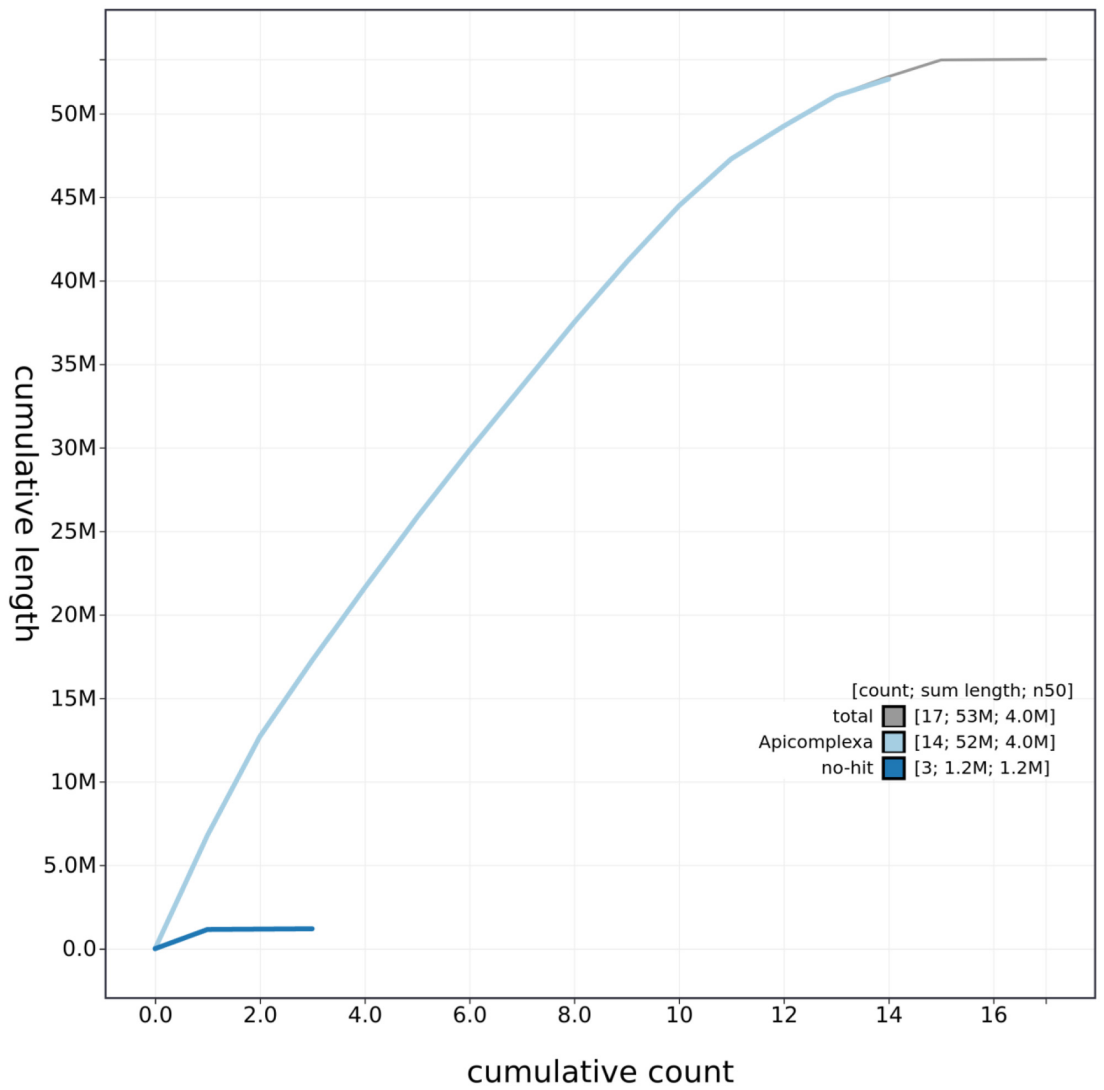
Genome assembly of *Eimeria tenella* Houghton, pEimTen1.1: cumulative sequence. BlobToolKit cumulative sequence plot. The grey line shows cumulative length for all chromosomes. Coloured lines show cumulative lengths of chromosomes assigned to each phylum using the buscogenes taxrule. An interactive version of this figure is available at https://blobtoolkit.genomehubs.org/view/pEimTen1.1/dataset/pEimTen1_1/cumulative.

**Table 2.** Chromosomal pseudomolecules in the genome assembly of *Eimeria tenella*, pEimTen1.1. The numbering of chromosomes is based on ordering the pEimTen1.1 assembly scaffolds by size in reverse order, so the chromosome names do not necessarily correspond to chromosome names in previously existing literature on *Eimeria tenella*.

INSDC accession	Chromosome	Size (kb)	GC%	Gaps	Putative centromeric region (bp)
HG994961	1	998.4	50.0	0	837838-871939
HG994962	2	1,151.2	47.8	1	530700-562938
HG994963	3	1,819.2	50.5	1	1605419-1629071
HG994964	4	1,948.7	50.0	2	1130403-1164585
HG994965	5	2,810.7	52.0	2	2256341-2281694
HG994966	6	3,367.5	51.8	3	2700201-2728956
HG994967	7	3,616.8	50.8	9	1871503-1901473
HG994968	8	3,810.9	51.5	1	1320955-1355380
HG994969	9	3,854.2	51.8	2	2305986-2344056
HG994970	10	4,007.2	53.4	1	2379713-2394995
HG994971	11	4,218.1	51.4	4	747612-790704
HG994972	12	4,348.4	52.3	0	418148-444959
HG994973	13	4,564.6	53.0	7	830432-888266
HG994974	14	5,913.3	51.4	4	3126091-3200449
HG994975	15	6,779.9	51.7	9	346670-377612
HG994976	MT	6.2	35.0	0	N/A
HG994977	Apicoplast	34.8	20.5	0	N/A

**Figure 4.**
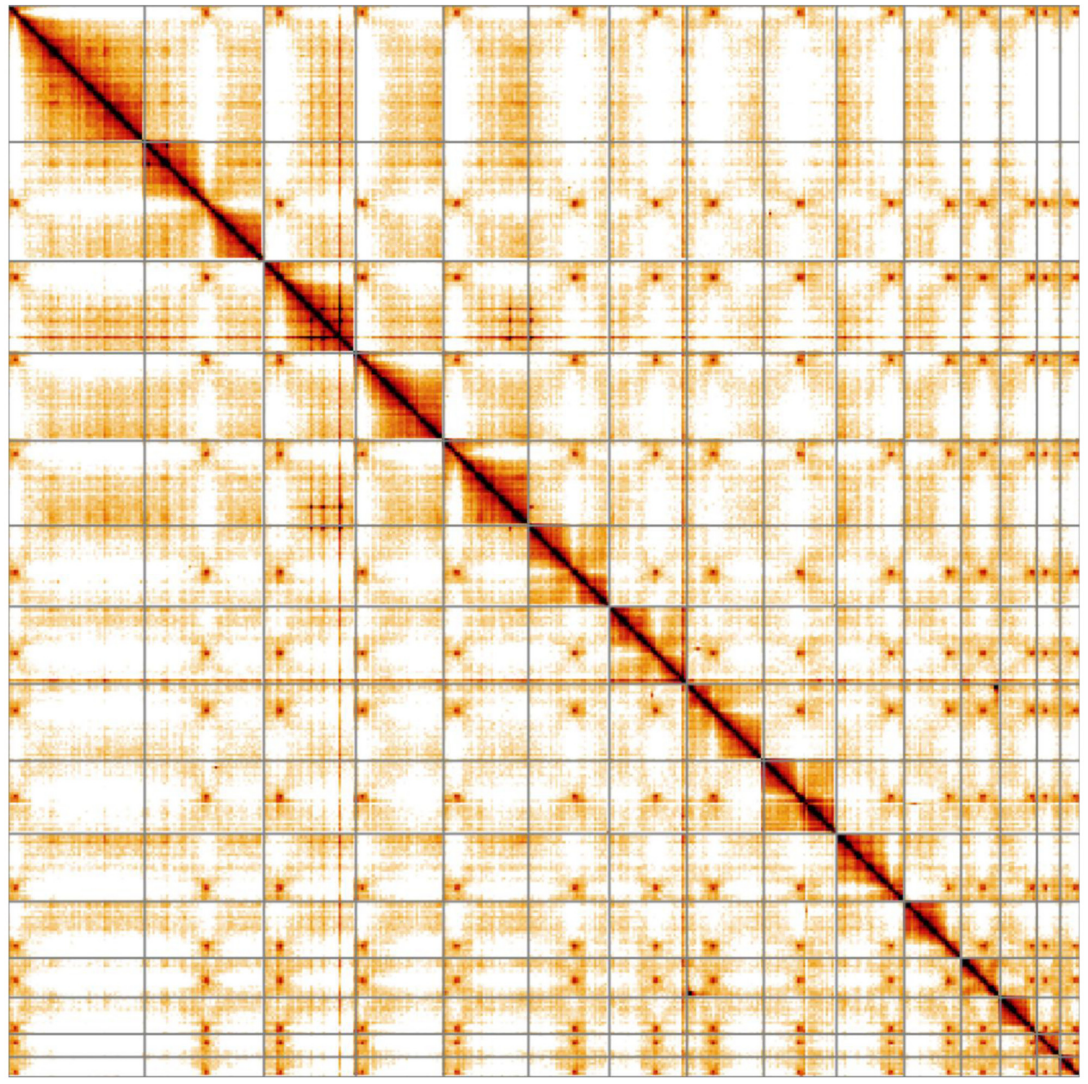
Genome assembly of *Eimeria tenella*, pEimTen1.1: Hi-C contact map. Hi-C contact map of the pEimTen1.1 assembly, visualised in HiGlass.

Of particular note is that 15 chromosomal scaffolds were identified, each with telomeres attached to both ends. This calls into question previous reports which suggested a haploid chromosome number of 14 for this species ( del Cacho *et al*., 2005). The Hi-C map ( Figure 4) shows that each of the 15 chromosomal scaffolds has a single contact region with each of the others. It has been shown in the coccidian relative *Toxoplasma gondii* that centromeres are sequestered together within the nucleus throughout the cell cycle ( Brooks *et al*., 2011). The Hi-C map suggests that this also occurs in *E. tenella* and if true, further supports the existence of 15 chromosomes. We examined the putative centromeric regions as identified by Hi-C in the Artemis genome browser ( Carver *et al*., 2012) and found almost all to be in intergenic regions of, on average, 35 kb (min=15 kb, max=74 kb). The exception was chromosome 1, where it was adjacent to a repeat near to the end of the chromosome. The data suggest that *E. tenella* chromosomes have single, well-localised centromeres which occupy acrocentric and sub-metacentric positions ( Table 2).

The GC content of the genome was 58.6%.

## Genome annotation report

We identified 7268 protein coding genes. Around 2000 gene models were manually corrected. The average exon length was 350.1, average intron length 298.1, with an average of 6.34 exons per gene. We annotated 44 pseudogenes, 32 degraded LTR retrotransposons (currently not included in GFF annotation), 140 rRNAs, 31 repeat regions, 28 ncRNAs and 345 tRNAs.

## Methods

A clonal specimen of *E. tenella* was collected from experimentally infected *G. gallus domesticus* at the Royal Veterinary College, Hertfordshire, UK. Four-week-old Lohmann Valo chickens reared under specific pathogen-free conditions were used to propagate oocysts of the *E. tenella* Houghton strain as described previously ( Long *et al*., 1976). Standard methods were used to purify and sporulate oocysts and to purify sporozoites through nylon wool and DE-52 columns ( Pastor-Fernández *et al*., 2019; Shirley *et al*., 1995). Animals were raised in strict accordance with the Animals (Scientific Procedures) Act 1986, an Act of Parliament of the United Kingdom. All animal studies and protocols were approved by the Royal Veterinary College Animal Welfare & Ethical Review Body (AWERB) and the UK Government Home Office under specific project licence.

DNA was extracted from the clonal specimen using the Qiagen MagAttract HMW DNA kit according to the manufacturer’s instructions. Pacific Biosciences CLR long read and 10X Genomics read cloud sequencing libraries were constructed according to the manufacturers’ instructions. Hi-C data were generated using the Arima Hi-C kit. Sequencing was performed by the Scientific Operations DNA Pipelines at the Wellcome Sanger Institute on Pacific Biosciences SEQUEL I (long read), Illumina HiSeq (10X) and Illumina MiSeq (Hi-C) instruments.

The assembly pEimTen1.1 is based on 41x PacBio data, 10X Genomics Chromium data, and Arima Hi-C data generated by the Darwin Tree of Life Project. PacBio subreads were assembled with Canu 1.6 ( Koren *et al*., 2017). After running Canu, some deduplication of contigs was performed using GAP5 v1.2.14-r3753M ( Bonfield & Whitwham, 2010). The assembly was scaffolded with scaff10x 4.2 using *E. tenella* 10x Chromium Illumina reads. This was then broken with break10x 3.1 and re-scaffolded using SALSA2 (October 2019 version) ( Ghurye *et al*., 2019) and *E. tenella* Hi-C reads. Juicebox 1.9.1 ( Robinson *et al*., 2018) and Tigmint 1.1.2 ( Jackman *et al*., 2018) were used to break scaffolds. RaGOO 1.1 ( Alonge *et al*., 2019) was then used to re-scaffold, using another assembly generated from the same PacBio reads using wtdbg2 2.5 (20190621) ( Ruan & Li, 2020). The assembly was then polished with Arrow (gcpp 1.0.0-SL-release-8.0.0, with pbmm2 version 1.1.0). Further polishing of the assembly was done with Pilon 1.19 ( Walker *et al*., 2014), using 10x Chromium Illumina reads from which 10x bar codes and linkers had been removed. The assembly was checked for contamination and analysed using the gEVAL system ( Chow *et al*., 2016) as described previously ( Howe *et al*., 2021). Manual curation was performed using gEVAL, HiGlass ( Kerpedjiev *et al*., 2018) and Pretext, before final polishing with Pilon. The genome was analysed and BUSCO v5.1.2 scores generated using BlobToolKit 2.6.1 ( Challis *et al*., 2020). The software tools used, with versions, are summarised in Table 3.

**Table 3.** Software tools used.

Software tool	Version	Source
Canu	1.6	( Koren *et al*., 2017)
GAP5	v1.2.14-r3753M	( Bonfield & Whitwham, 2010)
scaff10x	4.2	https://github.com/wtsi-hpag/Scaff10X
break10x	3.1	https://github.com/wtsi-hpag/Scaff10X
SALSA2	October 2019	( Ghurye *et al*., 2019)
Juicebox	1.9.1	( Durand *et al*., 2016)
Tigmint	1.1.2	( Jackman *et al*., 2018)
RaGOO	1.1	( Alonge *et al*., 2019)
Wtdbg2	2.5 (20190621)	( Ruan & Li, 2020)
Arrow	gcpp 1.0.0-SL-release-8.0.0	https://github.com/PacificBiosciences/GenomicConsensus
Pilon	1.19	( Walker *et al*., 2014)
STAR	2.5.3a	( Dobin *et al*., 2013)
Cufflinks	2.2.1	( Trapnell *et al*., 2010)
HISAT2	2.2.0	( Kim *et al*., 2019)
Companion	May 2020	( Steinbiss *et al*., 2016)
gEVAL	N/A	( Chow *et al*., 2016)
HiGlass	1.11.8	( Kerpedjiev *et al*., 2018)
PretextView	0.1	https://github.com/wtsi-hpag/PretextView
BlobToolKit	2.6.1	( Challis *et al*., 2020)

An initial annotation was performed using Companion ( Steinbiss *et al*., 2016) with the previous *Eimeria tenella* strain Houghton assembly and annotation as the reference ( Ling *et al*., 2007). *Eimeria tenella* RNA-seq reads (from project PRJEB3308 in the European Nucleotide Archive, runs ERR178634, ERR178635, ERR178636, ERR178637 and ERR178638 ( Reid *et al*., 2014)) were mapped to the assembly using 2-pass mapping method with STAR RNA-seq aligner version 2.5.3a ( Aunin *et al*., 2020; Dobin *et al*., 2013). The mapped reads were processed with Cufflinks v2.2.1 ( Trapnell *et al*., 2010) to produce a GTF file, which was then used as an input for Companion. Companion (May 2020 version) was run with Augustus threshold set to 0.2, alignment of proteins to the target genome enabled and other settings left as default. The annotations were then manually curated using Artemis v18.1.0 ( Rutherford *et al*., 2000) and the Artemis Comparison Tool v18.1.0 ( Carver *et al*., 2005) with the help of previously published RNA-seq data ( Reid *et al*., 2014). For viewing in Artemis, the RNA-seq data ( Reid *et al*., 2014) was mapped to the assembly with HISAT2 2.2.0 ( Kim *et al*., 2019).

## Data availability

European Nucleotide Archive: Eimeria tenella (Coccidian parasite). Accession number PRJEB43184: https://identifiers.org/ena.embl:PRJEB43184

The genome sequence is released openly for reuse. The *E. tenella* genome sequencing initiative is part of the Darwin Tree of Life (DToL) project. All raw sequence data and the assembly have been deposited in INSDC databases. Raw data and assembly accession identifiers are reported in Table 1.
