# Supplementary material for: The complete genome sequence of Eimeria tenella (Tyzzer 1929), a common gut parasite of chickens
Source: Wellcome Open Res. 2021 Sep 9;6:225. [Version 1] doi: 10.12688/wellcomeopenres.17100.1 (PMC8515493; doi:10.12688/wellcomeopenres.17100.1)
Supplement: Peer review history

# Peer review: Reviewer response for version 1

**DOI:** 10.21956/wellcomeopenres.18887.r45920

**Published:** 2021-10-13

**Authors:** Jon Boyle

**Affiliations:** 1 University of Pittsburgh, Pittsburgh, PA, USA

This is a nice genome assembly and is presented well by experts in the field. Hybrid assemblies are the norm now and the integration with Hi-C data is also impactful. 

 There are multiple software tools used and while versions are listed it would be very helpful if command line parameters were included in the manuscript so that these studies could be repeated by others either with the same data or with other similar data sets.

Are sufficient details of methods and materials provided to allow replication by others?

Partly

Is the rationale for creating the dataset(s) clearly described?

Yes

Are the datasets clearly presented in a useable and accessible format?

Yes

Are the protocols appropriate and is the work technically sound?

Yes

Reviewer Expertise:

genomics, toxoplasma gondii

I confirm that I have read this submission and believe that I have an appropriate level of expertise to confirm that it is of an acceptable scientific standard.

# Peer review: Reviewer response for version 1

**DOI:** 10.21956/wellcomeopenres.18887.r45921

**Published:** 2021-10-11

**Authors:** Arnab Pain

**Affiliations:** 1 Biological Environmental Sciences and Engineering (BESE) Division, King Abdullah University of Science and Technology (KAUST), Thuwal, Jeddah, 23955 6900, Saudi Arabia

The article (as Data Note) by Eerik Aunin and colleagues has produced a complete genome assembly and partially manually curated annotation of a clonal population of the apicomplexan parasite *Eimeria tenella* (Houghton strain) as part of the Darwin Tree of Life Project. *E. tenella* is a major parasite of chickens in the UK and worldwide and causes massive economic loss to poultry farming worldwide.

 Using a combination of single-molecule long reads (Pacific Biosciences), and 10X Genomics read clouds, they generated a set of primary assembly contigs which were subsequently scaffolded with the use of chromosome conformation Hi-C data. This resulted in 53.25 megabases assembly of the genome of *E. tenella* (Houghton), represented by 15 chromosomal pseudomolecules, along with complete organellar genomes (mitochondrion and apicoplast). Remarkably, the authors have provided sufficient evidence that *E. tenella* has 15 chromosomes (as opposed to 14 chromosomes reported previously) and all of them have telomeres attached to both ends – thus confirming the truly ‘complete’ nature of the assembly. The authors also re-annotated the genome with appropriate bioinformatics tools and with the use of bulk RNA-seq datasets generated as part of the original pan-Eimeria genome analysis study (Reid *et al*., 2014). The revised assembly has 7,268 protein-coding genes (out of which 2,000 were annotated manually) – reflecting an impressive BUSCO completeness of 98.8%.

   

 This is a truly remarkable achievement by the authors and I wish to congratulate them for this. Surely, access to this high-quality genome annotation and assembly will help researchers not only interested in coccidiosis in chicken but also in comparative genomics of apicomplexan parasites in general. I encourage the authors to also produce similar high-quality reference assemblies for the other *Eimeria* species as well (if supported by sufficient funding resources) that were previously reported in the *Eimeria* Pan-genomics study (Reid *et al*.). I encourage the authors to make this genome also publicly available via VEuPathDB for wider accessibility.

 The manuscript is clearly written with all the relevant details of all the tools used to generate the datasets and then analyze the results. I have absolutely no criticism for this manuscript. However, I have the following suggestions for the authors to add to the existing manuscript:

1. A comparative overview of how this assembly outperformed the previously assembled and annotated (& published) genome of *E. tenella* Houghton.
2. Since this represents a complete end-to-end assembly of all 15 chromosomes, a revised list of gene family annotations (such as the SAGs) would be very useful for the scientific community.

Are sufficient details of methods and materials provided to allow replication by others?

Yes

Is the rationale for creating the dataset(s) clearly described?

Yes

Are the datasets clearly presented in a useable and accessible format?

Yes

Are the protocols appropriate and is the work technically sound?

Yes

Reviewer Expertise:

Comparative genomics of apicomplexan parasites; Host-pathogen interactions, metagenomics-driven pathogen discovery.

I confirm that I have read this submission and believe that I have an appropriate level of expertise to confirm that it is of an acceptable scientific standard.

## References

[1] : Genomic analysis of the causative agents of coccidiosis in domestic chickens. *Genome Res* .2014;24(10) : 10.1101/gr.168955.113 1676-85 10.1101/gr.168955.113 25015382PMC4199364
